# Data on the frequency and causes of icteric interference in clinical chemistry laboratory tests

**DOI:** 10.1016/j.dib.2021.107771

**Published:** 2021-12-30

**Authors:** Anna E. Merrill, Sandhya Mainali, Matthew D. Krasowski

**Affiliations:** a Department of Pathology, University of Iowa Hospitals and Clinics, 200 Hawkins Drive, C-671 GH, Iowa City, IA 52242, USA; b Carver College of Medicine, University of Iowa, 451 Newton Road, Iowa City, IA 52242, USA; c Department of Psychiatry and Behavioral Sciences, University of Kansas School of Medicine-Wichita, Wichita, KS 67214, USA

**Keywords:** Alcoholic liver disease, Biliary tract diseases, Bilirubin, Clinical chemistry tests, Jaundice, Viral hepatitis

## Abstract

The presence of elevated levels of bilirubin (icterus) in serum or plasma specimens has the potential to interfere with clinical chemistry and other laboratory assays. Along with hemolysis and lipemia, icterus represents one of the most common endogenous interferences with laboratory tests. There are two common mechanisms by which icterus can cause assay interference. The first common mechanism is spectral interference due to absorption at wavelengths used in assays by bilirubin and/or bilirubin breakdown products. The second common mechanism involves chemical reaction of bilirubin with the reagents used in some enzymatic assays. Most automated clinical chemistry platforms can perform rapid estimates of indices for hemolysis, icterus, and lipemia (HIL), typically by measuring absorbance at wavelengths impacted by these interferences. The data in this article provides results from a detailed 12-month retrospective review of icteric indices and the impact on 114 clinical chemistry assays at an academic medical center in the United States. The data include 414,502 specimens from 94,081 unique patients (51,851 females; 42,230 males), with a total of 2,791,591 discrete clinical chemistry assays performed on the specimens. Detailed chart review was performed for all patients who had one or more specimens with an icteric index of 40 or higher (‘severe icterus’), including determination of the medical diagnoses likely causing icterus and the mortality of these patients within 1 and 3 years following laboratory testing. Data for all specimens include patient location at time of testing (emergency department, inpatient unit, or outpatient site), sex, age, HIL indices, specific clinical chemistry assays performed, and number of times specimens had icteric indices exceeding the icteric index threshold in the package inserts for the clinical chemistry assays performed. The dataset reported is related to the research entitled “Frequency of Icteric Interference in Clinical Chemistry Laboratory Tests and Causes of Severe Icterus” [S. Mainali, A.E. Merrill, M.D. Krasowski, Frequency of icteric interference in clinical chemistry laboratory tests and causes of severe icterus, Pract. Lab. Med. (2021) 27: e00259]

## Specifications Table

**Table:** 

Subject	Medicine and Dentistry
Specific subject area	Pathology and Medical Technology
Type of data	Tables
How data were acquired	Retrospective chart and data review from laboratory analysis performed at an academic medical center central clinical laboratory were obtained via tools within the electronic medical record.
Data format	Raw and Analyzed
Parameters for data were collection	Retrospective data on all clinical chemistry tests performed on a Roche Diagnostics cobas 8000 platform were obtained from the electronic medical record (Epic, Inc.) and laboratory middleware system covering the time period from January 1, 2018 through December 31, 2018. Detailed chart review was performed for the subset of patients who had one or more specimens with an icteric index of 40 or higher. The project had approval as a retrospective study from the University of Iowa Institutional Review Board (protocol # 201907707) with waiver of research subject informed consent.
Description of data collection	There were a total of 414,502 specimens from 94,081 unique patients (51,851 females and 42,230 males) during the retrospective analysis period. A total of 2,791,591 discrete clinical chemistry assays (including components of panels such as the basic metabolic panel) were performed on the specimens. The data collection from patient data contained laboratory test performed, patient sex (as recorded in electronic medical record), age in years at time of laboratory testing, patient location at time of testing (emergency department, inpatient unit, or outpatient clinic), hemolysis index of specimen, icteric index of specimen, and lipemic index of specimen. For patients with one or more specimens with an icteric index of 40 or higher, detailed chart review was performed for the likely medical diagnoses underlying the high level of icterus and whether patient was deceased within 1 year or 3 years of having a specimen with icteric index of 40 or higher. Compiled data for each clinical chemistry assay included assay name, assay vendor, package insert version relevant to study, package insert icteric index limit (if stated), total number of assays performed during retrospective timeframe, and number of assay results from specimens whose icteric index exceeeded the package insert icteric index limit for assays performed on that specimen.
Data source location	Iowa City, Iowa, United States of America
Data accessibility	Data identification number: 10.17632/vxkkdmw55f.1 Direct URL to data: https://data.mendeley.com//datasets/vxkkdmw55f/1
Related research article	S. Mainali, A.E. Merrill, M.D. Krasowski, Frequency of icteric interference in clinical chemistry laboratory tests and causes of severe icterus, Pract. Lab. Med. 27 (2021) e00259. https://doi.org/10.1016/j.plabm.2021.e00259

## Value of the Data

• The data provided are of value as icteric interference is one of the common endogenous interferences impacting clinical laboratory tests.
• Clinicians, other researchers, or personnel in clinical laboratories might find this data useful as a reference for comparison.
• The data set also contains data on hemolysis and lipemic indices, allowing for study of interactions between these interferences and icterus.
• The data provide information on icteric interference at an academic medical center performing high volumes of emergency department, inpatient unit, and outpatient clinic laboratory testing.
• The data provide information for 2,791,591 discrete assays performed on 414,502 specimens from 94,081 unique patients.

## 1 Data Description

Interferences in clinical laboratory specimens account for a significant source of error [1], [2], [3], [4], [5]. The most common endogenous interferences are hemolysis, icterus, and lipemia (HIL) [1,[6], [7], [8]. Icterus results from the presence of elevated concentrations of bilirubin (either conjugated or unconjugated or both) from a variety of different disease conditions [9,10]. Icterus can interfere with the clinical laboratory analysis by either spectral interference or chemical reactivity with assay reagents [4,^11^,12]. Automated clinical chemistry platforms typically offer the ability to determine 'indices' for HIL [3], [4], [5], [6],13]. This is generally done by diluting the specimen in buffer or saline and then measuring specific wavelengths interfered with by HIL.

This retrospective study includes data on 414,502 specimens from 94,081 unique patients (51,851 females and 42,230 males) for which clinical chemistry testing was performed at an academic medical center. Detailed chart review was performed for all patients who had at least one specimen with an icteric index of 40 or higher ('severe icterus').

The raw data for the study are included in Supplementary files 1–4.

• Supplementary file 1: Data for age in years (at time of laboratory testing) and sex (as recorded in the electronic medical record) for 94,081 unique patients. Ages greater than 89 years old are indicated as ">89". The retrospective timeframe is January 1, 2018 through December 31, 2018.
• Supplementary file 2: Data for 414,502 specimens submitted for clinical chemistry analysis. The retrospective timeframe is January 1, 2018 through December 31, 2018. Specific data fields include: unique specimen identification number (deidentified), patient location at time of testing (emergency department, inpatient unit, or outpatient site), sex (as recorded in electronic medical record), age in years (at time of laboratory testing), specimen hemolysis index, specimen icteric index, and specimen lipemic index.
• Supplementary file 3: Data for 114 clinical chemistry assays analyzed in the present study. Specific data fields include: assay name, assay vendor, package insert version relevant to the retrospective timeframe, assay package insert icteric index limit (if stated), total numbers of the specific test performed in the retrospective analysis, absolute number and percent of total specimen icteric indices that exceeded the package insert icteric index limit for the specific assay, and number of results that exceeded the package insert icteric index for a specific assay accounted for by 57 patients (4 pediatric, 53 adult; 524 total specimens) who had one or more icteric indices of 40 or higher in the retrospective timeframe. The retrospective timeframe is January 1, 2018 through December 31, 2018.
• Supplementary file 4: Data obtained by chart review for 57 patients (4 pediatric, 53 adult; 524 total specimens) who had one or more specimens with an icteric index of 40 or higher. The retrospective timeframe is January 1, 2018 through December 31, 2018. Specific data fields include: de-identified patient number, age in years (at time of laboratory testing), sex (as recorded in electronic medical record), diagnosis category from chart review that most likely accounted for severe icterus (grouped into broad categories of alcohol-related liver disease, biliary tract disease, liver disease related to infection, neoplasm/tumor, and other), additional/specific diagnoses, whether patient was deceased within 1 year of laboratory testing, and whether patient was deceased within 3 years of laboratory testing.

## 2 Experimental Design, Materials and Methods

### 2.1 Data source

The retrospective timeframe is January 1, 2018 through December 31, 2018. All data was obtained from patient data in the electronic medical record from the University of Iowa Hospitals and Clinics (Iowa City, Iowa, United States). A reporting tool within the electronic medical record, known as Epic Reporting Workbench [14], was used to retrieve data for 114 clinical chemistry tests performed in the retrospective timeframe. Only data from patients who had clinical chemistry testing performed at the University of Iowa Hospitals and Clinics were included; no data was obtained from diagnostic vendors of any of the laboratory assays used for clinical testing. HIL indices were obtained from Middleware software (Instrument Manager) from Data Innovations (Burlington, VA). Specimen accession numbers were used to link patient data, laboratory testing results, and HIL indices [15].

### 2.2 Analytical methology

The clinical chemistry instrumentation was a cobas 8000 system with two c702, three c502, and five e602 analyzers (Roche diagnostics, Indianapolis, IN, USA). The present study includes data for 105 assays using Roche Diagnostics reagents and 9 assays using reagents from other manufacturers that were run on the Roche cobas 8000 instrumentation. All serum/plasma specimens had HIL indices determined by spectrophotometry, which were then used for autoverification rules [16]. The analyzers take an aliquot of the patient specimen and dilute in 0.9% sodium chloride saline to measure the absorbance (primary/secondary wavelength) for hemolysis at 570 nm/600 nm, icterus at 480 nm/505 nm, and lipemia at 660 nm/700 nm. Detailed chart review was performed for 57 patients who had one or more specimens with an icteric index of 40 or higher.

## Ethics Statement

The analyses had approval by the University of Iowa Institutional Review Board (protocol #201907707) as a retrospective project with waiver of informed consent.

## CRediT authorship contribution statement

**Anna E. Merrill:** Formal analysis, Conceptualization, Writing – original draft, Writing – review & editing, Methodology. **Sandhya Mainali:** Formal analysis, Writing – review & editing. **Matthew D. Krasowski:** Formal analysis, Conceptualization, Writing – original draft, Writing – review & editing, Methodology, Supervision.

## Declaration of Competing Interest

The authors declare that they have no known competing financial interests or personal relationships that could have appeared to influence the work reported in this paper.
